# A cost-effectiveness evaluation of a high-sensitivity troponin I guided voluntary cardiovascular risk assessment program for asymptomatic women in Croatia

**DOI:** 10.1016/j.ijcrp.2024.200244

**Published:** 2024-02-10

**Authors:** Goran Krstačić, Paul Jülicher, Antonija Krstačić, Christos Varounis

**Affiliations:** a Institute for Cardiovascular Prevention and Rehabilitation (Srčana), Zagreb, Croatia; b J. J. Strossmayer University of Osijek Faculty of Dental Medicine and Health, Osijek, Croatia; c J. J. Strossmayer University of Osijek Faculty of Medicine, Osijek, Croatia; d Medical Affairs, Core Diagnostics, Abbott, Abbott Park, IL, USA; e University Hospital Center Sisters of Mercy, Zagreb, Croatia

**Keywords:** High-sensitivity troponin I, Biomarker, Risk assessment, Primary prevention, Cardiovascular disease, Cost-effectiveness

## Abstract

**Background:**

To estimate the effectiveness and cost-effectiveness of a high-sensitivity troponin I (hsTnI) guided cardiovascular risk assessment program in women in Croatia.

**Methods:**

An observational study of a voluntary program for cardiovascular disease (CVD) risk assessment in women aged above 45 years with no specific symptoms, no confirmed or known coronary artery disease was conducted (WHP). Participants were stratified into three categories according to their hsTnI level. Subjects in the moderate or high-risk class were referred to cardiac work-up and invasive cardiovascular investigation as appropriate. Study information were applied to a discrete-event simulation model to estimate the cost-effectiveness of WHP against current practice. The number of CVD events and deaths, costs, and quality-adjusted life years (QALY) were assessed over 10 years from a societal perspective.

**Results:**

Of 1034 women who participated in the program, 921 (89.1%), 100 (9.7%), and 13 (1.3%) subjects fall into the low, moderate, and high-risk class. Of 26 women referred for angiography, significant coronary artery disease (CAD) was diagnosed in 12 women (46.1%). WHP gained 15.8 (95%CI 12.8; 17.2) QALYs per 1000 subjects, increased costs by 490€ (95%CI 487; 500), decreased CVD-related mortality by 40%. At a willingness-to-pay threshold of 45,000 €/QALY, WHP was cost-effective with a probability of 90%. Model results were most sensitive to utility weights and cost of medical prevention.

**Conclusions:**

Assessing the cardiovascular risk in asymptomatic women with hsTnI and guiding those at higher risk to further cardiac testing, identified individuals with CAD, could reduce CVD related burden, and would be cost-effective.

## Highlights

• Elevated high-sensitivity troponin I (hsTnI) values are associated with higher rates of incident cardiovascular (CV) events.
• A hsTnI guided risk assessment program for apparently healthy women can identify CV disease and guide those at high risk towards prevention.
• This strategy can help reduce CV disease related burden and mortality and is likely to be cost-effective in Croatia.

## 1 Introduction

Cardiovascular disease (CVD) is a major contributor to the worldwide health burden and the number one cause of death globally [1]. The total economic burden of CVD in the EU was estimated to €210 billion in 2015 with 53% and 21% accounted for by direct medical costs and productivity losses [2]. In Croatia, although mortality from CVD has decreased over the last decade, CVD remains the leading cause of death and accounts for 48% of deaths in women and 37% in men [3,4]. Among these, coronary artery disease (or ischemic heart disease) is responsible for more than a third of all CVD related deaths [4]. As the general mortality rate from CVD is higher in women than in men, CVD in women have received special interest in the last ten years [4]. Due to a wide distribution of behavioral risk factors in Croatia including tobacco smoking and low physical activity, the mortality from preventable causes in Croatia is above the EU average [5]. With a share of almost 13%, CVD is the most frequent reason for hospitalization in Croatia with an average length of hospital stay of 7.2 days for men and 7.8 days for women [4]. To decrease the burden of CVD, reliable tools are needed to identify persons without known CVD who are at high cardiovascular risk and to guide those persons to lifestyle modifications or preventive medication [[6], [7], [8]]. Several risk assessment strategies have been recommended and are partly established [8]. Most of which are based on various risk algorithms derived from large cohort studies, such as the Framingham Risk Score, Q-Risk, or the recently updated SCORE (Systematic Coronary Risk Evaluation) risk calculator [[9], [10], [11], [12]]. The cardiac specific protein troponin I as measured using high sensitivity (hsTnI) assay methods is detectable in over 90% of the general population and has proven to be the most promising biomarker for determining individual cardiac risk when used in conjunction with existing clinical models [13,14]. It has been shown that elevated hsTnI values are associated with higher rates of incident fatal and non-fatal CV events, and act as an independent predictor for future CVD events [15,16]. Although hsTnI is being discussed for targeted primary prevention of asymptomatic individuals, a consensus on a uniform algorithm has not been proposed yet [14]. Despite the high burden of CVD in Croatia, a structured risk assessment program for CVD does not exist. The objective of our study is to test the effectiveness of a hsTnI guided risk assessment program in a voluntary women health program and estimate potential clinical and health economic consequences of applying this program at a national level in Croatia.

## 2 Methods

### 2.1 The Women & Heart Project Zagreb

Between January 2021 and December 2022, the Women & Heart Project (WHP) was implemented at the Institute for Cardiovascular Prevention and Rehabilitation (Srčana) in Zagreb as a voluntary program to assess cardiovascular risk in women aged above 45 years, with no specific symptoms and no confirmed or known coronary artery disease. All participants provided written informed consent an agreement in accordance with the General Data Protection Regulation of the EU and following the ethical principles outlined in the Declaration of Helsinki. Participants completed an online questionnaire about their principal characteristics, medical history, and self-reported awareness of cardiovascular risk factors. A blood sample was taken for the evaluation of laboratory parameters including hsTnI (ARCHITECT STAT High Sensitive Troponin-I, Abbott Laboratories, Abbott Park, Illinois, USA; Box S1). According to their hsTnI level, participants were stratified into three risk categories: Low-risk (hsTnI <4 ng/L), moderate-risk (hsTnI ≥4 – ≤10 ng/L), or high-risk category (hsTnI>10 ng/L). Subjects at low risk were discharged home without further intervention or follow-up. All subjects in the moderate and high-risk class were referred for non-invasive cardiac workup that consisted of examination by a cardiologist, electrocardiogram (ECG), echocardiography, exercise and 24-hourECG. If the left ventricular ejection fraction on the echocardiography was less than 50%, or a ST-segment depression (≥1 mm) was observed in the exercise ECG or 24 h ECG test, subjects were referred for coronary angiography. In subjects with significant obstructive coronary disease (narrowing ≥50% of coronary arteries) or high-risk plaques, percutaneous coronary intervention was recommended and subsequently performed. Subjects in the high and moderate hsTnI category with no findings in coronary angiography were considered for preventive treatment (lifestyle changes and high-dose statin plus ezetimibe).

### 2.2 Principal model design

**Fig. 1:**
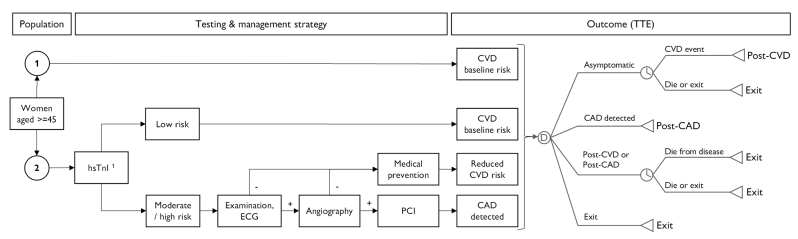
Comparing strategies. TTE: Time to event. CVD: Cardiovascular disease. hsTnI: High-sensitive troponin I. CAD: Coronary artery disease. WHP: Women & Heart Project Zagreb. Strategy 1: Current practice. Strategy 2: WHP strategy. Low hsTnI risk category: hsTnI <4 ng/L; Moderate hsTnI risk category: hsTnI ≥4 – ≤10 ng/L; High hsTnI risk category: hsTnI>10 ng/L.

A previously published discrete-event microsimulation model was adapted to the WHP [17]. The model compared two strategies in terms of the incidence of cardiovascular events from a societal perspective of Croatia. There is no general primary prevention program for CVD in current practice. Therefore, it was assumed as a standard strategy that there is no risk assessment with subsequent preventive measures. In the WHP strategy, individuals were screened with hsTnI and assigned to risk categories for CVD by applying gender-specific diagnostic cutoffs [15]. Subjects were managed according to the WHP protocol. All sampled subjects entered the model in an apparently healthy condition. Individual attributes and characteristics were directly informed by the WHP cohort. In case of a non-fatal CVD event or after detecting a coronary artery disease (CAD) in the WHP strategy, individuals moved into a post-CVD or post-CAD state until they died either from disease or any other causes, or they exited the model after the end of the time horizon. The model assessed the number of CVD events and deaths, the quality-adjusted life years (QALY), and the direct and indirect costs over a time horizon of ten years. Strategies and the principal model structure is illustrated Fig. 1, input assumptions are summarized in Table 1. Details about model calculations, sampling, sensitivity analyses, statistics, and model validation are available in Box S3.

**Table 1:** Model variables and assumptions.

Variable	Base value	Low^a^	High^a^	Sampling (1st order - 2nd order)	Reference
Time horizon	10	n/a	n/a	n/a	
Age at baseline	individual	n/a	n/a	Bootstrapped - n/a	WHP cohort
High-sensitive troponin-I	individual	n/a	n/a	Bootstrapped – n/a	WHP cohort
SCORE-2	Individual	n/a	n/a	Bootstrapped – n/a	WHP cohort
Background mortality	Individual	n/a	n/a	Per individual	[28]
Gross Domestic product per employed person, 2021€	35,870	−10%	10%	n/a	[33]
Exchange rate (Kuna to Euro)	0.13331	n/a	n/a	n/a	[18]
Labor force participation (female), %	61.96	−10%	10%	n/a - Uniform	[33]
Unemployment rate (female), %	8.02	−10%	10%	n/a - Uniform	[33]
Retirement age	65	n/a	n/a	n/a	
Friction period in years	1	0.5	1.5	n/a	
Proportion not returned to work after CVD event, %	12.0	9.0	15.0	n/a - Beta	[34]
Reduction in productivity due to absenteeism after CVD event, %	1.4	0.5	2.5	n/a - Beta	[19,35,36]
Reduction in productivity due to presenteeism after CVD event, %	3.8	2.5	4.0	n/a - Beta	[19,35,36]
Invasive cardiac workup in subjects with hsTnI>10 ng/L, %	92.3	77.0	99.0	n/a - Beta	WHP cohort
Invasive cardiac workup in subjects with hsTnI 4–10 ng/L, %	14.0	7.0	21.0	n/a - Beta	WHP cohort
Risk functions, risk probabilities					
Standard risk function of individuals in the hsTnI low risk class: Weibull shape	1.235	1.103	1.383	Weibull - n/a	[17]
Standard risk function of individuals in the hsTnI moderate risk class: Weibull shape	1.158	1.033	1.298	Weibull - n/a	[17]
Standard risk function of individuals in the hsTnI high risk class: Weibull shape	0.954	0.816	1.114	Weibull - n/a	[17]
Standard risk function of individuals in the hsTnI low risk class: Weibull scale	179.30	132.51	242.60	Weibull - n/a	[17]
Standard risk function of individuals in the hsTnI moderate risk class: Weibull scale	58.97	48.31	72.00	Weibull - n/a	[17]
Standard risk function of individuals in the hsTnI high risk class: Weibull scale	32.86	25.91	41.67	Weibull - n/a	[17]
Hazard ration to adjust risk functions to Croatia	1.524	1.37	1.68	n/a - LogNormal	[17,20]
Hazard ratio of preventive measures	0.56	0.49	0.69	Beta - n/a	[21]
CVD fatality (age <55yrs), %	5.8	5.2	6.4	n/a - Beta	[25]
CVD fatality (age 55–74yrs), %	12.8	11.5	14.1	n/a - Beta	[25]
CVD fatality (age≥75yrs), %	29.8	26.8	32.8	n/a - Beta	[25]
5-year cumulative post-CVD mortality (Age<55, surviving >28 d), %	6.1	5.5	6.7	Exponential - Uniform	[25]
5-year cumulative post-CVD mortality (Age 55–74), %	19.7	17.7	21.7	Exponential - Uniform	[25]
5-year cumulative post-CVD mortality (Age 75–85), %	44.3	39.9	67.0	Exponential - Uniform	[25]
In-hospital mortality following PCI, %	1.9	1.7	2.1	n/a - Beta	[26]
5-year cumulative post-CAD mortality, %	4.0	3.6	4.4	Exponential - Uniform	[27]
Costs, 2021€					
Examination by cardiologist	12	−25%	+25%	n/a - Uniform	University Hospital Center
Standard laboratory	10	−25%	+25%	n/a - Uniform	Assumption
Incremental costs for hsTnI	25	−25%	+25%	n/a - Uniform	Assumption
Noninvasive cardiac tests (ECG)	140	−25%	+25%	n/a - Uniform	University Hospital Center
Invasive cardiac tests (Angiography)	1901	−25%	+25%	n/a - Uniform	University Hospital Center
Interventional cardiology (PCI)	2085	−25%	+25%	Gamma - n/a	University Hospital Center
Acute CVD event	4378	−25%	+25%	Gamma - n/a	[29]
Rehabilitation after CVD	1632	−25%	+25%	Gamma - n/a	[30]
Medical prevention (annual)	732	−25%	+25%	n/a - Uniform	[31]
Annual discount rate for costs, %	5.0	3.0	10.0	n/a	[37]
Utility weights					
Baseline	0.94	0.91	0.97	n/a	
Decrement under preventive medication	0.003 (0.05)	0.002	0.004	Beta – n/a	[22]
Decrement for PCI	0.06 (0.02)	0.00	0.10	Beta – n/a	[23]
Decrement for CVD event	0.13 (0.10)	0.05	0.42	Beta – n/a	[24]
Post-CVD	0.82 (0.17)	0.74	0.90	Beta – n/a	[24]
Post-CAD	0.91 (0.05)	0.88	0.95	Beta – n/a	[23]
Annual discount rate, %	5.0	3.0	10.0	n/a	[37]

CVD: Cardiovascular disease. hsTnI: High-sensitivity troponin I. PPP: Purchasing power parity.

a Boundaries used in univariate sensitivity analyses.

### 2.3 Risk functions and time-to-event

Standard risk functions stratified by hsTnI risk categories were described by Weibull distributions and were retrieved from a previously described study [17]. The model was adopted to the National level of Croatia by adjusting standard risk functions to age and sex specific incidence data for Croatia as described in Box S2 [20]. The time to CVD event were sampled per each individual by risk category from respective Weibull distributions as described previously [17, 21]. Fatality of CVD events were not available for Croatia and taken from a Dutch study [25]. In-hospital mortality following PCI were obtained from a US registry [26]. Post-CVD and post-CAD mortality were applied by sampling from exponential distributions considering a constant rate based on five-year cumulative probabilities [25,27]. Background mortality were estimated from country specific life tables by considering the age at CVD event [28].

### 2.4 Costs, utilities, and outcomes

Effectiveness of strategies were measured in terms of CVD events, CVD deaths, and quality-adjusted life years (QALY), According to the underlying study for estimating the standard risk, CVD referred to a composite endpoint of hospitalization for acute myocardial infarction or heart failure, or cardiovascular death [15]. Direct medical costs included expenditures for testing, preventive medication, non-invasive and invasive cardiac workup, interventional cardiology, and costs for CVD hospitalization. Costs for cardiac workup and interventional cardiology were taken from the study site and determined by the Croatian Health Insurance Institute. Costs for hospitalization and rehabilitation after acute CVD in Croatia were obtained from the literature [29,30]. Annual costs for medical prevention medication were derived from a Croatian drug utilization study [31]. For patient management after an event, annual medication costs were assumed supplemented by the cost of one (CAD) or two (CVD) cardiologist visits. If required, all costs were adjusted for inflation by using the GDP implicit price deflator and reported as 2021 Euro [18,32,33]. Indirect costs were assessed from CVD related productivity losses in the working population and took workplace absenteeism, presenteeism, reduced employment, and lost productivity due to premature death into account. The number of fatal events before the retirement age were adjusted with the labor force participation and unemployment rate. To calculate the loss in productivity associated with premature death, this product was multiplied with the GPD per employed person assuming a friction period of one year to replace the worker. The proportion of employees who did not return to work after a CVD event were assessed from a Dutch survey following employed patients [34]. Productivity costs associated with premature death or reduced employment assumed a friction period of one year. The reductions in productivity due to absence from work (absenteeism) and reduced work performance (presenteeism) were both derived from US-studies [19,35,36] and were applied to the working years after a non-fatal event to the end of the model horizon multiplied by the GDP per person employed. Utility values for Croatia were not available and were therefore obtained from literature [[22], [23], [24]]. Future costs and benefits were discounted into a present value with a fixed discount rate of 5% [37]. Cost-effectiveness was discussed by comparing the incremental cost-effectiveness ratio (ICER) to the willingness-to-pay threshold for cost-effective strategies suggested by the World Health Organization (3 times the GDP per capita. Croatia: €45,000) [38].

## 3 Results

### 3.1 Women & Heart Project Zagreb

**Fig. 2:**
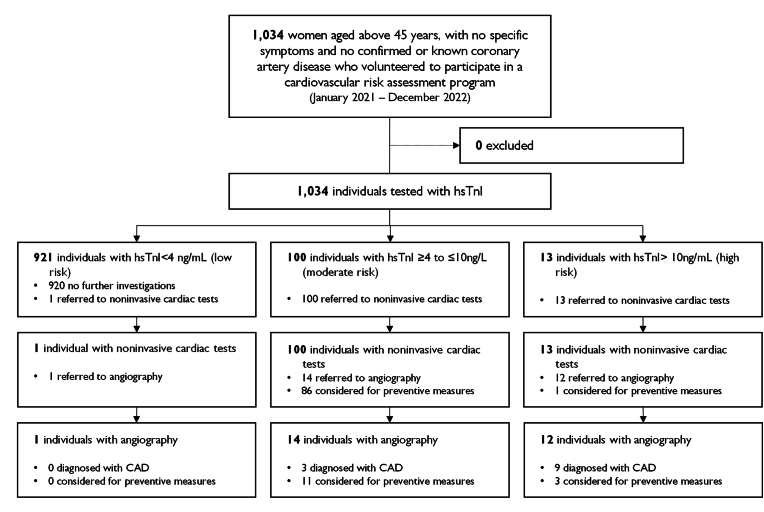
Flowchart of the WHP risk assessment program. WHP: Women & Heart program Zagreb. CVD: Cardiovascular disease. hsTnI: High-sensitive troponin I. CAD: Coronary artery disease.

A total of 1034 women voluntarily agreed to participate in the WHP. Characteristics, self-reported cardiovascular risk factors and laboratory results are shown in Table 2 and Table S1. Mean age at baseline was 55.6 (interquartile range 49.0–62.0) years. Based on the online questionnaire, the prevalence of smokers was 25.0%, 26.6% had hypertension, 45.9% dyslipidemia, and 7.3% diabetes. Depending on the hsTnI-results, 921 (89.1%), 100 (9.7%), or 13 (1.3%) subjects were classified as low, moderate, and high risk, respectively. Compared to the low hsTnI risk category, subjects at moderate and high risk were older, had a higher total cholesterol level, a higher LDL level, more frequently reported a family history of CVD or a known diabetes (Table 2; Table S1; Table S3). All subjects at moderate and high risk were assessed with non-invasive cardiac tests (n = 113, 10.9% of all). Of these, 26 (2.51% of all) were further referred to invasive cardiac tests, of which 14 and 12 subjects fall into the moderate and high hsTnI risk category, respectively. Despite classified as low risk, one woman was referred to cardiac workup but was found negative in coronary angiography. A total of 12 (1.16% of all) subjects were eventually diagnosed with CAD, three (3.0% of women at moderate risk) and nine (69.2% of women at high risk) of whom were categorized in the moderate or high troponin risk class, respectively (Fig. 2, Table S5).

**Table 2:** Cohort characteristics.

Patient characteristics		Proportions, %			
	N	All	RC 1	RC 2	RC 3
All	1034	100	89.1	9.7	1.3
Age <50 years	278	26.9	91.7	8.3	0.0
Age 50–69 years	703	68	89	9.2	1.7
Age≥70 years	53	5.1	75.5	22.6	1.9
Family history^a^	1034	19.4	15.7	47.0	69.2
Known diabetes^a^	1034	7.3	6.4	13.0	23.1
Dyslipidemia^a^	1034	45.9	45.7	45.0	69.2
Arterial hypertension^a^	1033	26.6	26.1	34.3	7.7
Smoker^a^	1034	25	25.4	23.0	7.7
Physical Activity^a^	1034	50.4	50.5	50.0	46.2
Previous COVID-19 infection^a^	1034	23	22.8	26.0	15.4
SARS-COV2 vaccination^a^	1033	28.1	27.4	36.0	15.4
Total cholesterol > 5 mmol/L	1034	72.7	70.8	88.0	92.3
LDL >3 mmol/L	1034	73.2	71.4	88.0	84.6
HDL <1.2 mmol/L	1034	9.6	8.8	16.0	15.4
Triglycerides >1.7 mmol/L	1034	15.2	14.7	18.0	30.8
HbA1c >6%	1034	14.9	14	24.0	7.7
hsCRP >3 mg/L	1034	25.7	23.7	44.0	30.8

a Self-reported online-questionnaire. RC1: hsTnI <4 ng/L (low risk); RC2: hsTnI ≥4 – ≤10 ng/L (moderate risk); RC3: hsTnI >10 ng/L (high risk). hsTnI: high-sensitivity troponin-I.

### 3.2 Cost-effectiveness model results

**Fig. 3:**
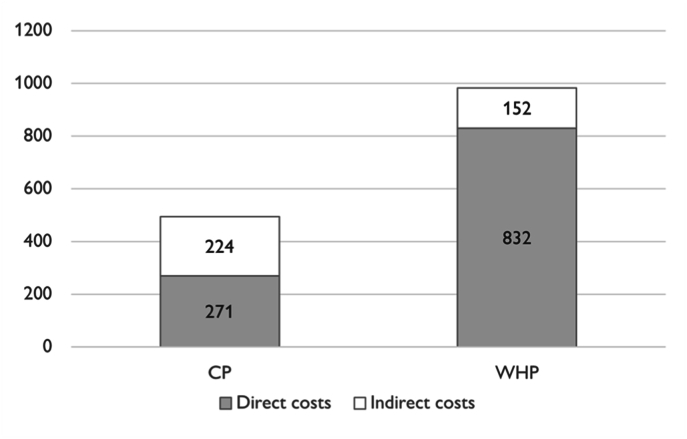
Direct and indirect medical costs. Comparing strategies: CP: Current practice. WHP: Women & Heart Project. Costs in 2021 Euro.

Results of the model calibration to the National level in Croatia are presented in Box S4. Results of the microsimulation analysis are summarized in Table 3. In the standard strategy, 429 (95%CI 411; 445) CVD events and 89 (81; 91) CVD related deaths occurred per 10,000 subjects over ten years. In the WHP strategy, 53 (95%CI 46–56) per 10,000 screened women were diagnosed with CAD during the workup process. Compared to current practice, the incidence of CVD events was reduced by 180 per 10,000 (95%CI 177; 184) resulting in a 40% decrease of CVD related deaths. The average number of women screened to prevent one CVD event in a period of ten years was 56. The reduced disease burden led to a gain of 15.5 (95%CI 12.8; 17.2) additional QALYs per 1000 subjects. While the WHP strategy increased direct medical cost from 271€ to 832€ per subject (+561€, 95%CI: 544; 576), indirect cost was reduced by 32% or 72€ (95%CI 59; 84). Total cost per subject was increased by 489€ (95%CI 487; 500) (Fig. 3). In 97% of iterations of the MS the resulting ICER was below the threshold of 45,000€ indicating a high probability that WHP is cost-effective compared to CP (Table S10). Cost-effectiveness acceptability curve showed that for willingness-to pay thresholds above 30,000€ per QALY, the WHP strategy became the preferred strategy with 90% probability at a WTP of 45,000€ (Fig. S2). Univariate sensitivity analyses revealed that apart from the standard risk functions, model results are most sensitive to the utility weights and the cost of medical prevention (Fig. S3, Fig. S4). More than 40% of the total uncertainty is represented by the health state utility weights and decrements. Results were found stable in probabilistic sensitivity analyses (PSA) (Table S9), but also indicated a high variability in the average QALY gain while the difference in prevented CVD events was robust (Fig. S5, Fig. S6).

**Table 3:** Cost-effectiveness microsimulation results.

Outcome	CP		WHP		Difference	
	Mean	95%CI	Mean	95%CI	Mean	95%CI
Costs, €	495	(481; 518)	983	(975; 1011)	489	(487; 500)
QALY, x1,000	7023	(7015; 7031)	7038	(7029; 7047)	15.5	(12.8; 17.2)
ICER, € per QALY					31,555	(25,802; 45,663)
CVD, per 10,000	429	(417; 445)	249	(238; 260)	−180	(-184; −177)
CVD deaths, per 10,000	89	(82; 91)	53	(48; 56)	−36	(-37; −34)

CP: Strategy reflecting current practice. WHP: Strategy reflecting the Women & Heart Project protocol. QALY: Quality adjusted life-years. ICER: Incremental costs per incremental QALY. Confidence intervals derived from results of 100 independent iterations of the microsimulation.

## 4 Discussion

Best to our knowledge, the WHP is the first study that provides prospective observational data for using high-sensitivity troponin I for CVD risk stratification in an asymptomatic population. More than 1000 women in Zagreb volunteered to participate in the study. Guided by the use of the biomarker hsTnI, participants were excluded or referred to cardiac testing and interventional cardiology, if required. More than 89% of enrolled women fall into the low-risk class for CVD and were excluded from further workup. In 10.6% of subjects referred to further workup, significant CAD was identified and managed, likely preventing acute cardiac event and/or CVD related death. This study confirmed the association between the hsTnI level and the cardiovascular outcome in asymptomatic women. In a trial-based cost-effectiveness model, the WHP strategy was extrapolated to the national level in Croatia. Compared to current practice, WHP reduced the number of CVD events and related deaths by 42% and 40%, respectively, which has a clear public health impact. In summary, this resulted in a statistically significant increase of 15.5 QALYs per 1000 women over a period of 10 years. Testing women with the WHP protocol increased direct medical costs, but decreased indirect costs caused by CVD related productivity loss. WHP was cost-effective with a probability of 90% and 80% at cost-effectiveness thresholds of 45,000€ and 39,000.

As the WHP was a non-controlled trial, the baseline risk of subjects had to be estimated. Given the huge variation of CVD risk between countries [2], the previously described risk functions could not be used to simulate the baseline risk in Croatia directly [17]. Instead, we corrected the standard risk functions by using a hazard ratio of 1.542 that was derived from iterative calibration. According to the European Society of Cardiology, Croatia is regarded as high-risk country and an adjustment factor of 1.643 has been suggested to adjust the recently updated SCORE to high-risk regions [12,39]. The factor is slightly higher than the calibration factor that we determined (Box S4). However, this value is not country-specific but for a high-risk region and, secondly, based on a different basis for the calculation. According to our sensitivity analysis (Fig. S3), a higher factor would further favor the WHP strategy.

As suggested by the CAD detection rate, the WHP cohort was at higher risk compared to the Croatian estimate used in the model (1.12% vs. 0.53%). Statistical significance cannot be clearly demonstrated, and the difference can be due to a very small number of cases for patients in the hsTnI risk category (Table S8). This could be supported by reports that mortality from CVD differs between regions in Croatia but are slightly below the national average in Zagreb [4]. Nevertheless, a real difference in risk between the participating women and the national average cannot be excluded: The WHP was a voluntary program and women provided information in a self-reported survey prior the first laboratory test, and the accuracy and quality of cohort characteristics could not be assessed. A study suggests that voluntary screening programs tend to underestimate the disease prevalence [40]. Whether the women who participated in the WHP are representative of the population or whether a selection bias has to be considered, is not clear. Since cost-effectiveness of the WHP strategy increases with risk, however, the results can be considered conservative.

Current risk assessment tools have several limitations such as a restricted age range or the difficulty of applying such tools in regions with different baseline risk for CVD [41]. Some limitations have been addressed by the development of the updated SCORE2 to estimate 10-year CVD risk in Europe which is also recommended by the guidelines on CVD prevention by the European Society of Cardiology (ESC) [12,39]. However, the use of risk assessment tools is highly variable, and there is still a lack of evidence in the overall effectiveness and cost-effectiveness of risk assessment tools [8,[42], [43], [44]]. Consequently, the implementation of a uniform risk assessment tool including SCORE into routine practice is largely insufficient [8]. Several articles have demonstrated and discussed the usefulness of hsTnI for CVD risk assessment [14]. The most efficient algorithm for using hsTnI for CVD risk assessment is still subject for discussion [14], and only one article is proposing a potential algorithm [45]. In this premature situation, the WHP has been developed as a biomarker guided risk assessment program for women to identify those with high individual cardiac risk. Albeit 54% of all CVD events occurred in subjects with hsTnI < 4 ng/L who account for 89.1% of the total population, testing with hsTnI effectively stratifies subjects by their individual cardiac risk. With increasing hsTnI value, the cumulative 10-years risk for CVD increased from 2.6% to 16.7% and 37.2% in the low risk, moderate risk, and high hsTnI risk class, respectively. Further cardiac workup of subjects with elevated hsTnI led to diagnosis of established CAD and prevented acute CVD events and deaths. Guiding subjects with negative cardiac test results to preventive measures helps to reduce the incidence of future events. Therefore, the resulting NNS of 56 to avoid one acute CVD event through the WHP program is a combined result of assessing the risk, intensive cardiac workup, and long-term prevention management.

Our study is based on an adapted version of a previously published model which estimated the cost-effectiveness of using hsTnI for assessing CVD risk in a general population [17]. As in our study, subjects from a hypothetical cohort were stratified according to their hsTnI level and those at high risk were referred to preventive means. Compared to a do-nothing approach in 1000 subjects in Germany, the hsTnI guided strategy reduced the number of CVD events by five, equal to a NNS of 191, and gained 13.9 QALYs over a period of ten years. The strategy was found cost-effective with an ICER of $6755 per QALY (2018 International Dollar). Unlike our study, more subjects were classified as moderate risk (18%) and high risk (4%). As the cost-effectiveness ratio becomes less favorable with decreasing prevalence of subjects with elevated hsTnI, one should expect the ICER with WHP to be worse. However, in the WHP study, subjects were first referred to further cardiac testing, which, as a result, led to a higher reduction of acute CVD events, but also higher costs. Nevertheless, the overall result indicates a high probability that this investment is still cost-effective.

On average, following the WHP protocol required 93€ per subject and a further 559€ for persons with no CAD referred to medical prevention. Thus, 11% of the total direct medical costs per person were spent on screening and testing and 67% for medical prevention over ten years (Table S13). According to the WHP protocol, a broad range of non-invasive and invasive cardiac tests were considered in order to confirm diagnoses and refer women to more intensive management (Table S6). Adopting the WHP protocol to clinical practice will most likely limit the use of these tests to what is necessary. This would reduce direct medical costs required for the WHP protocol thus further strengthening its cost-effectiveness.

Specific utility weights for CVD in Croatia were not available and therefore obtained from literature. However, studies demonstrated that utility weights for each CVD state varies widely. As indicated in the tornado diagram, uncertainty in health state utility weights substantially impacts the results and became apparent in the broad distribution of QALY results of the PSA (Fig. S5). Model results are much more stable with regard to the costs per CVD event prevented (Fig. S6). However, the translation of the average number of prevented CVD events to QALYs varies considerably between different microsimulation runs and ranged from 0,24 to 1.86 additional QALYs over a period of ten years (IQR: 0.71–1.09; Fig. S7). For more accurate assessment of the cost-effectiveness of the WHP strategy, a more precise determination of quality-of-life weights is needed.

Although recommended for assessing the cardiovascular risk, we did not consider SCORE2 in our analysis [39]. First, some characteristics critical for estimating SCORE2 were based on a self-reported questionnaire, were not validated or not available. An accurate calculation of SCORE2 was therefore not possible. Second, the size of the study was not adequate to explore the differences between hsTnI and SCORE2 in terms of predictive value. Third, SCORE2 has not been widely implemented into clinical practice in Croatia. This, however, points to an important area for future research.

We acknowledge several further limitations. Our study estimated the cost-effectiveness of applying the WHP strategy based on national figures and assumed a full coverage of the tested population, which is not realistic. This should be considered before interpreting potential effects on the national burden of CVD. As discussed, the model was not based on a controlled study or national figures, and the actual underlying CVD risk of the tested cohort compared to Croatian data as well as the distribution of the female population by hsTnI risk class is unknown. We only considered the first CVD event and did not take potential events into account that may occur after detecting CAD. Both aspects likely have an impact on all tested strategies and should be considered to finally assess the value of a new strategy. For CVD risk reduction in our model, we only used a mean value that was informed by the efficacy of statin treatment but did not further address the actual prevention measure. There are several preventive measures, ranging from life-style advice to high-intensive medication. Besides the effectiveness of drugs, systematic studies demonstrated the efficacy of life-style interventions on CVD risk factors as well [46]. Sensitivity analyses show that the HR of the preventive measure does not substantially affect the model results either. Therefore, we consider this simplification to be appropriate in the context of our study. Life tables were not adjusted for CVD risk and may therefore overestimate the background mortality. This effect may have limited the value of the WHP strategy. All results should be interpreted against these limitations. Future studies should evaluate specific hsTnI risk assessment algorithms in conjunction with and in comparison, to established risk assessment tool, as well as the potential economic impact of serial hsTnI measurements.

## 5 Conclusions

Assessing the cardiovascular risk in asymptomatic women with hsTnI and guiding those at higher risk to further cardiac testing, identified individuals with CAD and referred those at high risk to preventive measures. This strategy could reduce CVD related burden and mortality and would likely be cost-effective in Croatia. More studies are needed to confirm these findings.

## Funding

The Women and Heart Project is funded by the City of Zagreb, City Office for Social Protection, Health, Veterans and Persons with Disabilities, Activity A211124R. There is no sponsorship or funding to declare from Abbott.

## Data availability statement

As restrictions apply, patient-level data underlying this article are not publicly available. Other data of this study are available upon reasonable request to the corresponding author.

## CRediT authorship contribution statement

**Goran Krstačić:** Conceptualization, Data curation, Investigation, Methodology, Resources, Supervision, Validation, Writing – review & editing. **Paul Jülicher:** Conceptualization, Formal analysis, Methodology, Software, Validation, Visualization, Writing – original draft, Investigation. **Antonija Krstačić:** Conceptualization, Methodology, Writing – review & editing. **Christos Varounis:** Conceptualization, Validation, Writing – review & editing.

## Declaration of competing interest

PJ and CV are full-time employees of Abbott. GK and AK report no relationships that could be construed as a conflict of interest.
