# Supplementary material for: A cost-effectiveness evaluation of a high-sensitivity troponin I guided voluntary cardiovascular risk assessment program for asymptomatic women in Croatia
Source: Int J Cardiol Cardiovasc Risk Prev. 2024 Feb 10;20:200244. doi: 10.1016/j.ijcrp.2024.200244 (PMC10928367; doi:10.1016/j.ijcrp.2024.200244)
Supplement: Multimedia component 1 [file mmc1.pdf]

Supplementary Material

A cost-effectiveness evaluation of a high-sensitivity troponin I guided voluntary cardiovascular risk assessment program for asymptomatic women in Croatia

Goran Krstačić, Paul Jülicher, Antonija Krstačić, Christos Varounis

Abbreviations

CP	Current practice
CVD	Cardiovascular disease
ESC	European Society of Cardiology
HR	Hazard ratio
hsTnI	High sensitivity troponin I
ICER	Incremental cost-effectiveness ratio
NNS	Number needed to screen
QALY	Quality-adjusted life year
WHP	Women & Heart Project Zagreb
WTP	Willingness to pay

Box S 1. High Sensitivity Troponin-I Measurement

Fasting blood samples were drawn and processed for plasma samples. Plasma hsTnI was measured by using ARCHITECT STAT High Sensitive Troponin-I (Abbott Laboratories, Abbott Park, Illinois, USA). The limit of detection of this test is 1.6 ng/L and 99th percentile at 15.6 ng/L for female with a coefficient of variance of 5.3%.[1]

Box S 2. Adjustment of baseline cardiovascular risk functions to Croatia

Standard risk functions stratified by hsTnI risk categories were described by Weibull distributions and were retrieved from a previously described study.[2] In order to adapt the model to the National level of Croatia, we collected age and sex specific incidence data for Croatia.[3] In a simplified version of the model, the occurrence and time of CVD events were compared between the standard risk functions and the GBD arm. In order to adjust the standard risk functions to Croatia, we made the following assumptions: (a) Differences in risks between countries are expressed as hazard ratios, which in the case Croatia is described by $HR_{CR} = (b_0 / b_{CR})^{\alpha}$, where b_0 and b_{CR} denote the Weibull scale parameter for the standard risk and for Croatia, respectively, and α represents the Weibull shape parameter; (b) the Weibull shape parameter for each hsTnI risk category is constant between countries; (c) the specific HR_{CR} for Croatia is constant between hsTnI risk classes. In a next step, we applied HR_{CR} to the standard risk functions and adjusted the standard risk functions by iterative calibration of HR_{CR} against the cumulative number of CVD events and healthy life years (HLY) after 10 years as estimated by the GBD arm. The results of the adjusted risk functions were statistically compared. The country specific HR for Croatia were applied to the standard risk functions to estimate the baseline risk for women in Croatia.

Box S 3. Model calculations, sampling, sensitivity analysis, statistics, and validation

The model was developed in TreeAge Pro 2022 (TreeAge Software, Williamstown, MA, USA) and in accordance to the CHEERS guidelines for good research practices in modelling and reporting.[4] All statistical analyses were performed in Minitab Statistical Software 19 (Minitab, LLC, State College, PA, USA). The decision-analytic analysis was performed using a first order Monte Carlo microsimulation of 100,000 individuals so that the pooled standard deviation of costs and QALYs of strategies in ten independent runs were lower than the mean difference between strategies. Individual characteristics were randomly sampled per each trial from the WHP cohort by bootstrapping or from respective distributions.

To reflect either heterogeneity or parameter uncertainty, variables were sampled from distributions either during microsimulation or as outer loops during probabilistic sensitivity analyses (PSA). Comparisons between strategies were made based on mean outcome values with confidence intervals derived from results of 100 independent runs of the microsimulation. For the incremental cost-effectiveness ratio (ICER), confidence intervals were estimated from the 2.5th and 97.5th percentile of the ICER distribution as obtained from 100 independent iterations of the microsimulation. Univariate sensitivity analyses were conducted on all variables by varying input values between the lower and upper bound as stated in Table 1. The impact on the incremental cost-effectiveness ratio were reported as tornado diagrams. PSA was performed by applying a second order Monte Carlo simulation of critical variables in 250 iterations of the microsimulation. Statistical significance was analyzed conducting a two-sample t-test with a significance level of 0.05.

Model validation was conducted in several steps.[5] Standard risk functions were validated in a previous study and calibrated to Croatia as described above.[2] Model structure and assumptions were informed by an extensive literature review.[6] Input assumptions, data sources, formulas and results were critically reviewed by experts. Individual trackers were used to capture individual outcomes and to validate model calculations. Model outcomes of the WHP strategy at the National level were compared with observational data of the WHP.

Box S 4. Model calibration

By iterative calibration to GBD incidence for women in Croatia, a risk factor HR_{CR} of 1.542 was calculated. Both outcomes, the number of CVD events and HLY, showed no statistical difference between the adjusted risk functions and the GBD estimate (Table S4). Also, a comparison of survival plots confirmed the results (Fig S1). Model results of the WHP strategy at the National level showed good agreement with observational data of the WHP between the data for low (p 1.0) and moderate (p 0.7) troponin risk category. However, the estimated number of CAD at the national level in the high-risk group was significantly lower (p 0.04) (Table S5).

Table S 1. Cohort characteristics by hsTnI risk category.

	ALL			RC1			RC2			RC3			p-value*
**Patient characteristics**	**Mean**	**Median**	**IQR**	**Mean**	**Median**	**IQR**	**Mean**	**Median**	**IQR**	**Mean**	**Median**	**IQR**	
Age, years	55.6	55	49 - 62	55.4	54	49 - 61	57.2	56.5	50 - 63	60.3	61	55 - 63	< 0.01
hsTnI, ng/L	2.1	1.3	1.3 - 2.0	1.7	1.3	1.3 - 2.0	4.9	4.0	4.0 - 6.0	11.7	11.0	10.0 - 11.5	< 0.01
Total cholesterol, mmol/L	5.8	5.7	5.0 - 6.5	5.7	5.6	4.9 - 6.4	6.4	6.6	5.8 - 7.2	7.1	7.1	5.6 - 8.4	< 0.01
LDL, mmol/L	3.6	3.6	3.0 - 4.2	3.6	3.5	3.0 - 4.1	4.0	4.0	3.5 - 4.6	4.4	4.6	3.3 - 4.9	< 0.01
HDL, mmol/L	1.6	1.6	1.4 - 1.8	1.6	1.6	1.4 - 1.8	2.0	1.3	0.4 - 1.6	3.0	1.4	0.5 - 1.6	0.88
Triglycerides, mmol/L	1.3	1.1	0.8 - 1.5	1.2	1.1	0.8 - 1.5	1.3	1.2	1.0 - 1.5	1.9	1.2	0.8 - 1.9	0.08
HbA1c, %	5.6	5.7	5.5 - 5.9	5.6	5.7	5.5 - 5.9	5.8	5.7	5.6 - 6.0	5.6	5.6	5.4 - 5.8	0.02
hsCRP, mg/L	2.5	1.5	0.7 - 3.1	2.4	1.3	0.7 - 2.8	2.9	2.3	1.2 - 4.5	3.8	1.6	1.0 - 6.1	0.03

hsTnI risk categories: RC1: hsTnI <4ng/L; RC2: hsTnI ≥4 – ≤10ng/L; RC3: hsTnI >10 ng/L. IQR: Interquartile range. hsTnI: high-sensitivity troponin I. *RC3 & RC2 vs. RC1.

Table S 2. Cohort characteristics by clinical outcome.

Variable	No CAD			CAD			p-value of the mean
	**Mean**	**Median**	**IQR**	**Mean**	**Median**	**IQR**	
Age, years	55.6	55.6	49 - 62	58.0	61.0	51 - 62	0.20
hTnI, ng/L	2.0	1.3	1.3 - 2.0	11.3	11.0	9.3 - 11.8	<0.01
Total cholesterol, mmol/L	5.8	5.8	5.0 - 6.5	7.3	7.3	6.1 - 8.3	<0.01
LDL, mmol/L	3.6	3.6	3.0 - 4.2	4.5	4.7	3.8 - 4.9	0.03
HDL, mmol/L	1.62	1.60	1.40 - 1.80	1.61	1.70	1.08 - 1.95	0.93
Triglycerides, mmol/L	1.3	1.2	0.8 - 1.5	1.9	1.1	0.9 - 1.8	0.28
HbA1c, %	5.6	5.7	5.5 - 5.9	5.7	5.7	5.5 - 5.8	0.63
hsCRP, mg/L	2.5	1.5	0.7 - 3.1	4.5	2.5	1.1 - 7.8	0.14

CAD: Coronary artery disease. IQR: Interquartile range. hsTnI: high-sensitivity troponin I

Table S 3. Self-reported characteristics by hsTnI risk category.

Patient characteristics	Proportions, %			
	All	RC1	RC2&3	p-value*
Family history	19.4	15.7	49.6	<0.01
Known diabetes	7.3	6.4	14.1	<0.01
Dyslipidemia	45.9	45.7	47.9	0.69
Arterial hypertension	26.6	26.1	31.3	0.26
Smoker	25	25.4	21.2	0.36
Physical Activity	50.4	50.5	49.6	0.92
Previous Covid infection	23	22.8	24.8	0.64
Covid vaccination	28.1	27.4	33.6	0.18

Self-reported questionnaire. hsTnI risk categories: RC1: hsTnI <4ng/L; RC2: hsTnI ≥4 – ≤10ng/L; RC3: hsTnI >10 ng/L. IQR: Interquartile range. hsTnI: high-sensitivity troponin I. *Test for two-proportions using Fisher's exact methodology.

Table S 4. Self-reported characteristics by clinical outcome.

Patient characteristics	Proportions, %			
	All	CAD	No CAD	p-value*
Family history	19.4	83.3	18.3	<0.01
Known diabetes	7.3	16.7	7.1	0.22
Dyslipidemia	45.9	50	45.9	0.78
Arterial hypertension	26.6	8.3	26.8	0.20
Smoker	25	8.3	25.2	0.31
Physical Activity	50.4	33.3	50.6	0.26
Previous Covid infection	23	16.7	23.1	0.99
Covid vaccination	28.1	16.7	282	0.53

*Test for two-proportions using Fisher's exact methodology.

Table S 5. Management of subjects participating in the Women & Heart Project Zagreb.

Troponin risk class (RC)	N	% of all	Non-invasive work-up		Invasive work-up		CAD		
			N	% of all	N	% in RC	N	% of RC	% of all
All	1034	100.0	114	11.03	27	2.61	12	1.16	1.16
Low*	921	89.1	1	0.10	1	0.11	0	0.00	0.00
Moderate	100	9.7	100	9.67	14	14.00	3	3.00	0.29
High	13	1.3	13	1.26	12	92.31	9	69.23	0.87

Troponin risk class: Low (hsTnI <4ng/L) Moderate (hsTnI ≥4 and ≤10ng/L) High (hsTnI >10ng/L). Non-invasive workup: examination by a cardiologist, electrocardiogram (ECG), echocardiography, exercise and 24-hourECG. Invasive workup: coronary angiography.

Table S 6. Direct medical costs for cardiac workup used in the model.

Item	2021 Euro
**Non-invasive cardiac workup**	**139.95**
Examination by a cardiologist	11.72
ECG at rest	5.55
24h blood pressure measurement	23.36
24 ECG	30.92
Echocardiography (1,2 D + doppler)	32.00
Exercise ECG test	36.40
**Invasive cardiac workup**	**1901.27**
MSCT coronarography	278.91
SPECT (nuclear cardiology)	293.10
Coronary angiography	1329.26
**Interventional cardiology**	**2085.24**
Stent	318.54
PCI balloon	515.63
Microwire	292.65
Lead wire	958.42

Fig S 1. Survival plot for CVD risk functions before and after adjustment to WHO incidence data for Croatia.

Red line: GBD Incidence Croatia 2019 (High risk country). Blue line: Summary survival plot based on risk functions (trop-risk class specific) derived from published KP plots (HUNT study) Country specific risk factor before adjustment HR_{CR} =1 (A) and after adjustment HR_{CR} =1.542 (B).

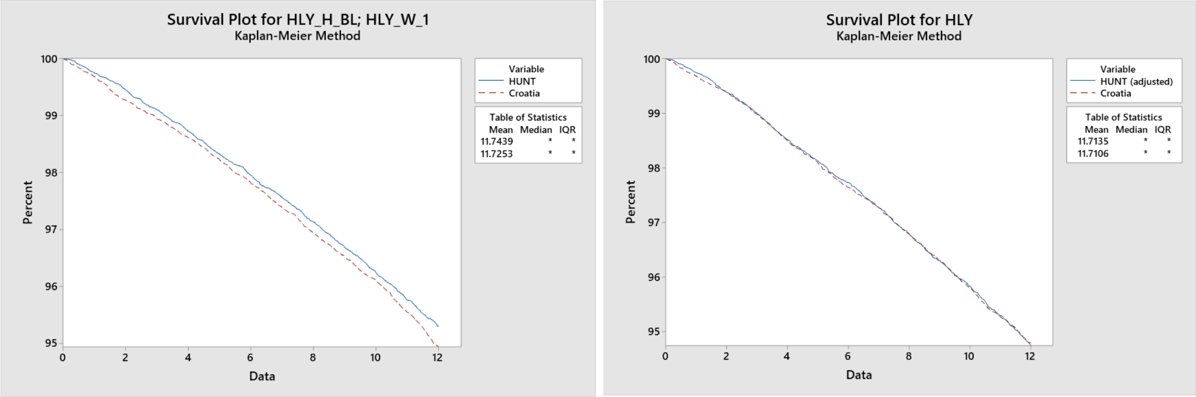

Table S 7. Survival plot for CVD risk functions before and after adjustment to WHO incidence data for Croatia.

(A) After adjustment

Variable	Mean	SE Mean	StDev	Difference	95% CI for Difference	p-value
CVD (Standard risk)	0.048	0.001	0.213			
CVD (Adjusted baseline risk)	0.048	0.001	0.215	-0.001	(-0.004; 0.003)	0.79
HLY (Standard risk)	10.85	0.017	2.68			
HLY (Adjusted baseline risk)	10.87	0.017	2.68	-0.015	(-0.062; 0.032)	0.53

CVD: CVD events. HLY: Healthy life years.

(B) Nonparametric Estimates

			95.0% Normal CI	
Model	Mean (MTTF)	SE	Lower	Upper
HUNT (adj.)	11.714	0.009	11.695	11.732
WHO	11.711	0.009	11.692	11.729

(C) Test statistics

Method	Chi-Square	DF	p-value
Log-Rank	0.0494	1	0.824
Wilcoxon	0.0496	1	0.824

Table S 8. Comparison of the WHP Cohort and the WHP Strategy in the Croatian Model.

hsTnI risk class (RC)	WHP Cohort			WHP strategy in the model			Difference	
	%	% CAD in RC	95%CI	%	% CAD in RC	95%CI	95%CI	p-value
**All**	100.0	1.2	(0.5 - 1.8)	100.0	0.5	(0.5 - 0.6)	(-1.28; 0.03)	0.05
Low	89.1	0.0	(0.0 - 0.0)	89.1	0.0	(0 - 0)	(0 - 0)	1.00
Moderate	9.7	3.0	(0.0 - 6.4)	10.1	2.4	(2.1 - 2.7)	(-2.77; 4.05)	0.71
High	1.3	69.2	(40.2 - 98.2)	0.8	37.7	(34.2 - 41.1)	(2.3; 60.8)	0.04

hsTnI: High-sensitivity troponin I. hsTnI risk class: Low (hsTnI <4ng/L) Moderate (hsTnI ≥4 and ≤10ng/L) High (hsTnI >10ng/L).

Table S 9. Probabilistic sensitivity analysis results.

Outcome	CP			WHP			Difference		
	MS	PSA		MS	PSA		MS	PSA	
		Mean	95%CI		Mean	95%CI	Mean	Mean	95%CI
Costs, €	493	505	(484 - 530)	983	993	(933 – 1,059)	491	488	(484; 493)
QALY, x1,000	7,022	7,020	(6,998 – 7,035)	7,038	7,037	(7,023 – 7,046)	16	17	17,496
CVD, per 10,000	429	434	(421 - 447)	249	250	(247 - 252)	-180	-184	(-185; -183)
CVD deaths, per 10,000	88	91	(79 - 103)	53	57	(49 - 63)	-35	-35	(-35; -34)
ICER, € per QALY							30,863	31,707	(17,496; 55,773)

MS: Microsimulation (n=100,000 trials). PSA: 250 runs of MS. ICER 95%CI estimated from the 2.5th and 97.5th percentile of the distribution of all results.

Table S 10. Proportion of incremental cost-effectiveness results per quadrant.

COMPONENT	QUADRANT	Incremental values			Proportion, %	
		QALY	Cost	ICER	MS	PSA
C1	IV	IE>0	IC<0	Superior	0.0	0.0
C2	I	IE>0	IC>0	ICER<45,000	97.0	90.4
C3	III	IE<0	IC<0	ICER>45,000	0.0	0.0
C4	I	IE>0	IC>0	ICER>45,000	3.0	10.0
C5	III	IE<0	IC<0	ICER<45,000	0.0	0.0
C6	II	IE<0	IC>0	Inferior	0.0	0.0
Indiff.	origin	IE=0	IC=0	0/0	0.0	0.0

MS: 100 iterations of the microsimulation (no outer loop). PSA: probabilistic sensitivity analysis with 250 iterations of the MS (incl. outer loop). IE: Incremental effectiveness. IC: Incremental costs. ICER: Incremental cost-effectiveness ratio.

Table S 11. Current practice and WHP.

hsTnI risk class	CP				WHP				
	All	%	CVD	Risk, %	WHPP	CAD	Prevention	CVD	Risk %
All	100,000	100.00	4,293	4.3	10,864	531	10,333	2,488	2.5
Low	89,136	89.14	2,315	2.6	0	0	0	2,315	2.6
Moderate	10,086	10.09	1,681	16.7	10,086	238	9,848	169	1.7
High	778	0.78	290	37.3	778	293	485	4	0.5

MS: Microsimulation (n=100,000 trials).

Table S 12.

hsTnI risk class	CP	WHP	ARR	RRR	NNS
**All**	0.043	0.025	0.018	0.420	56
Low	0.026	0.026	0.000	0.000	n/a
Moderate	0.167	0.017	0.150	0.901	7
High	0.373	0.005	0.368	0.986	3

MS: Microsimulation (n=100,000 trials). CP: Current practice. WHP: Women & Heart Project. ARR: Absolute risk reduction. RRR: Relative risk reduction. NNS: Number needed to screen.

Table S 13. Direct medical costs per subject.

Strategy	Direct Costs	WHP protocol	Prevention	CVD events & rehabilitation
CP	**271**	0	0	271
WHP	**832**	93	558	180
CP, undiscounted	**353**	0	0	353
WHP, undiscounted	**1030**	93	702	236

Microsimulation (n=100,000 trials). Comparing strategies: CP: Current practice. WHP: Women & Heart Project. All figures in 2021 Euro.

Fig S 2. Cost-effectiveness Acceptability Curve.

Probability for cost-effectiveness of WHP strategy. Probabilistic sensitivity analysis (250 iterations of the microsimulation).

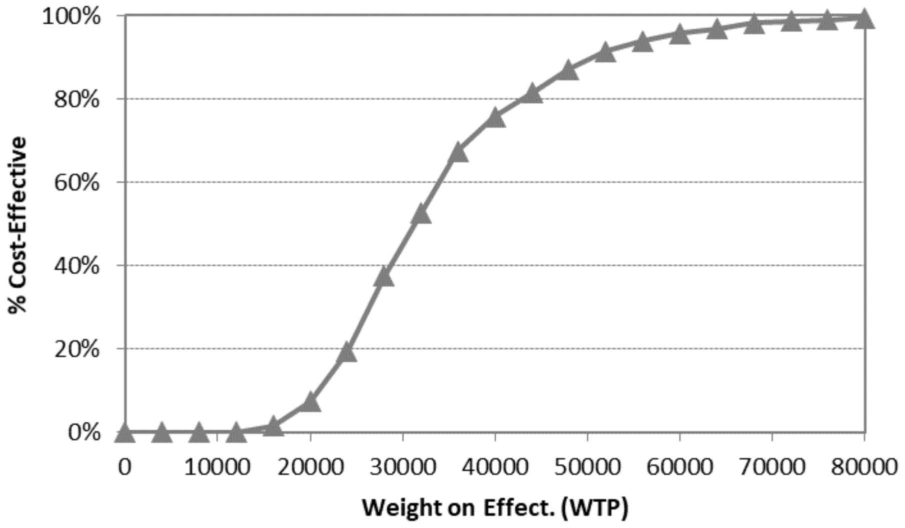

Fig S 3. One-way sensitivity analysis tornado diagram.

ICER: Incremental cost-effectiveness ratio. Results from multiple one-way sensitivity analyses for variables within the ranges stated in Table 1.

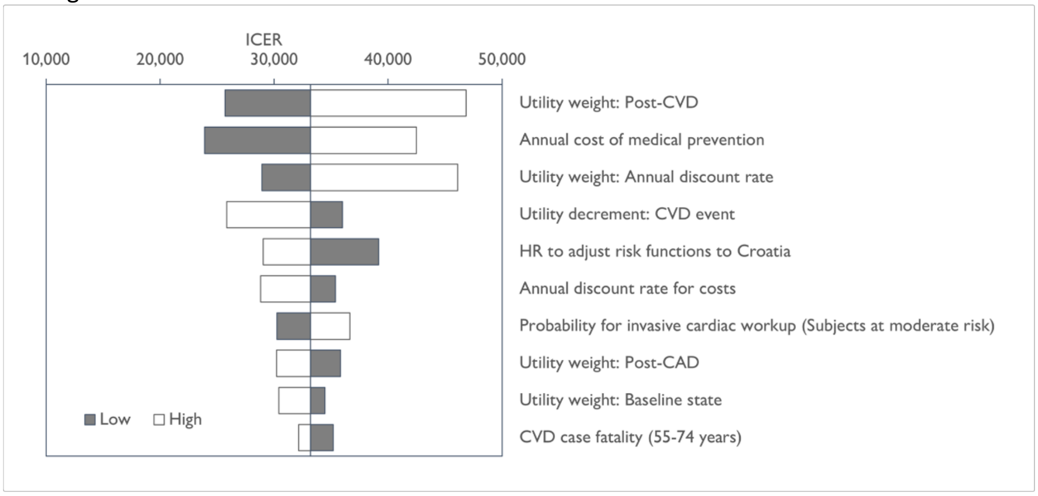

Fig S 4. One-way sensitivity analysis tornado diagram.

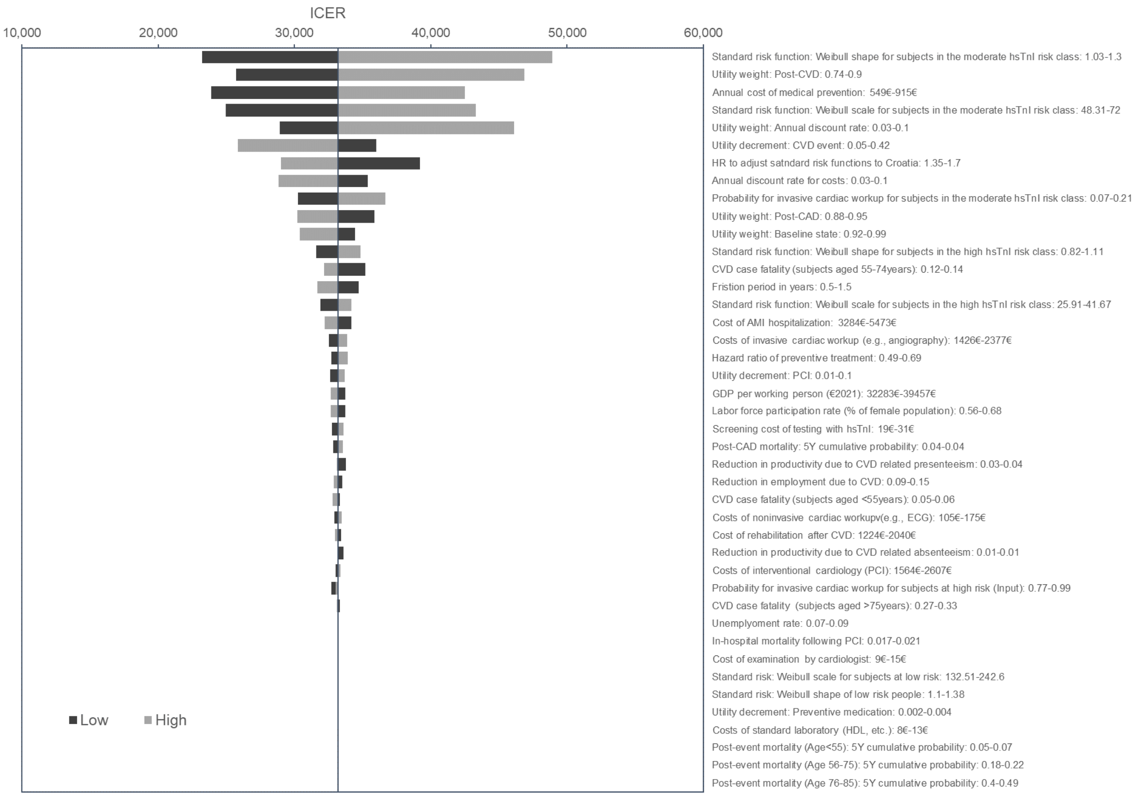

Fig S 5. Cost-effectiveness from probabilistic analysis.

Probabilistic sensitivity analysis (250 outer loops, 100,000 inner loops). Incremental cost and incremental QALY of current practice (Square) and the WHP strategy (Triangle). The dashed line corresponds to the results from the microsimulation.

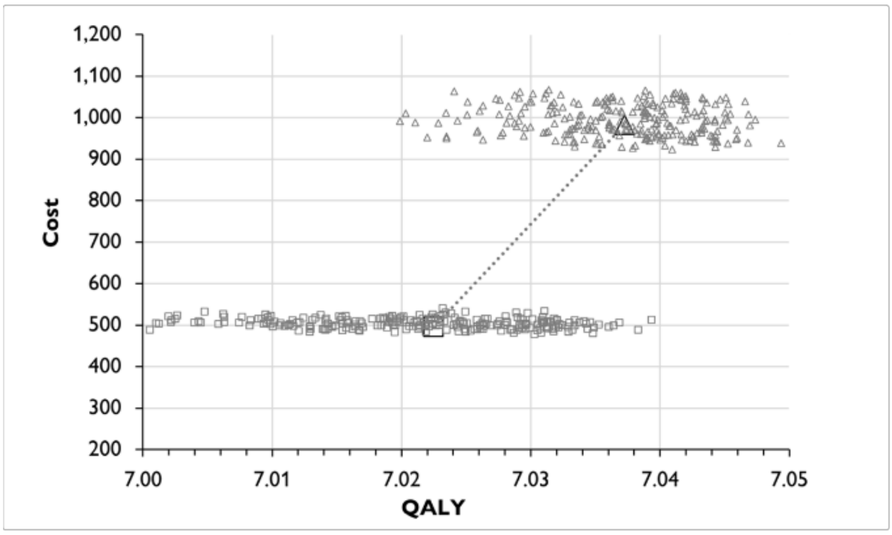

Fig S 6. Cost-effectiveness matrix cost vs. prevented CVD events.

Probabilistic sensitivity analysis (250 outer loops, 100,000 inner loops) results for the number of CVD events per 10,000 screened subjects and costs of current practice (□) and the WHP strategy (Δ).

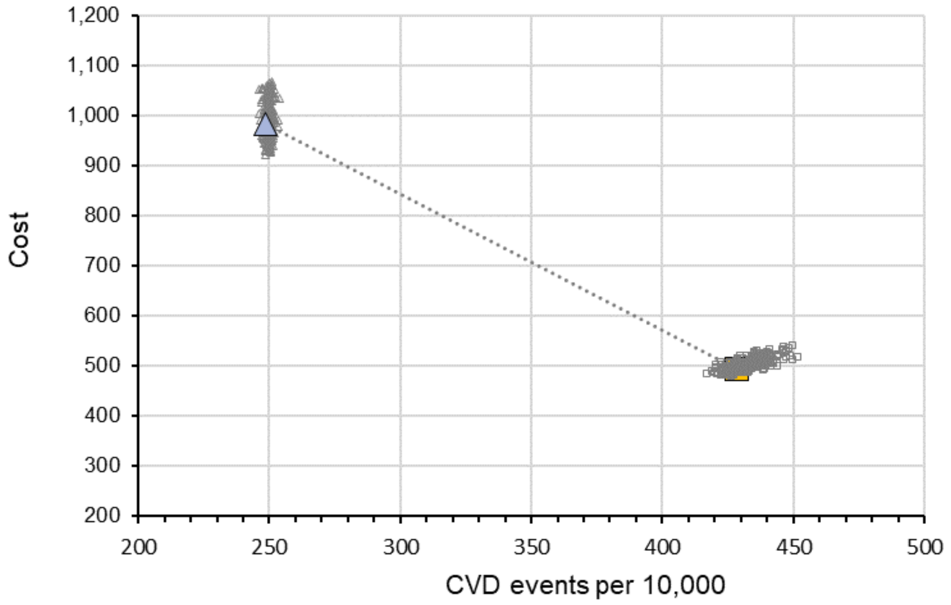

Fig S 7. Incremental QALY per prevented CVD for WHP strategy compared to CP.

Comparing strategies: CP: Current practice. WHP: Women & Heart Project. Each datapoint reflects the result of one iteration of the probabilistic sensitivity analysis. The dotted line represents the linear regression considering an intercept of zero. On average, one prevented CVD translated into 0.918 (IQR 0.714; 1.086) additional QALYs over a period of ten years.

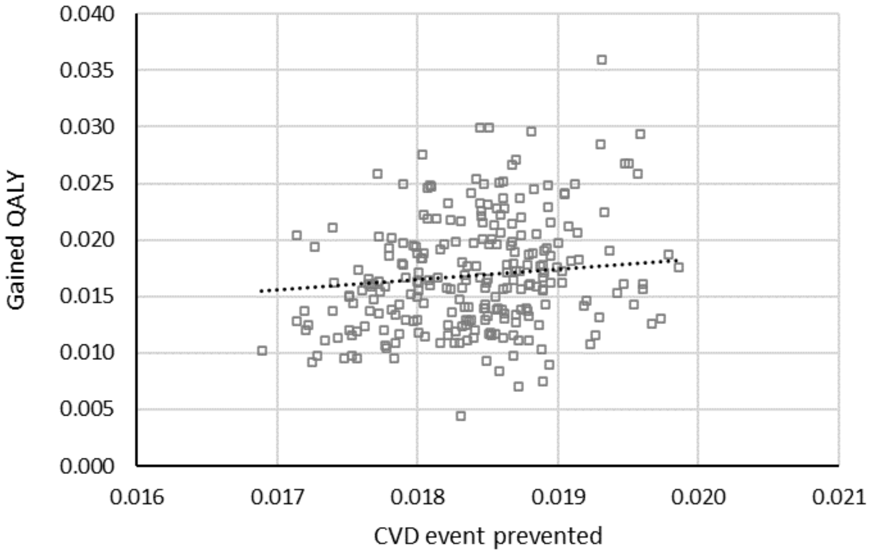

References

1. Committee on Clinical Applications of Cardiac Bio-Markers (The International Federation of Clinical Chemistry and Laboratory Medicine): Biomarkers Reference Tables. https://ifcc.org/ifcc-education-division/emd-committees/committee-on-clinical-applications-of-cardiac-bio-markers-c-cb/biomarkers-reference-tables/ (Aug 22 2022)
2. Julicher P, Varounis C. Estimating the cost-effectiveness of screening a general population for cardiovascular risk with high-sensitivity troponin-I. *Eur Heart J Qual Care Clin Outcomes* 2021. doi: 10.1093/ehjqcco/qcab005
3. Benjak T, Cerovečki I, Draušnik Ž, Fuštin D, Uhernik AI. [Results of the EUROSTAT project "Morbidity Statistics" Data for Croatia]. In: Capak K, (ed): Croatian Institute of Public Health; 2022.
4. Husereau D, Drummond M, Petrou S*, et al.* Consolidated Health Economic Evaluation Reporting Standards (CHEERS) statement. *Value Health* 2013;**16**:e1-5. doi: 10.1016/j.jval.2013.02.010
5. Eddy DM, Hollingworth W, Caro JJ*, et al.* Model transparency and validation: a report of the ISPOR-SMDM Modeling Good Research Practices Task Force-7. *Value Health* 2012;**15**:843-850. doi: 10.1016/j.jval.2012.04.012
6. Epstein D, Garcia-Mochon L, Kaptoge S, Thompson SG. Modeling the costs and long-term health benefits of screening the general population for risks of cardiovascular disease: a review of methods used in the literature. *Eur J Health Econ* 2016;**17**:1041-1053. doi: 10.1007/s10198-015-0753-2
